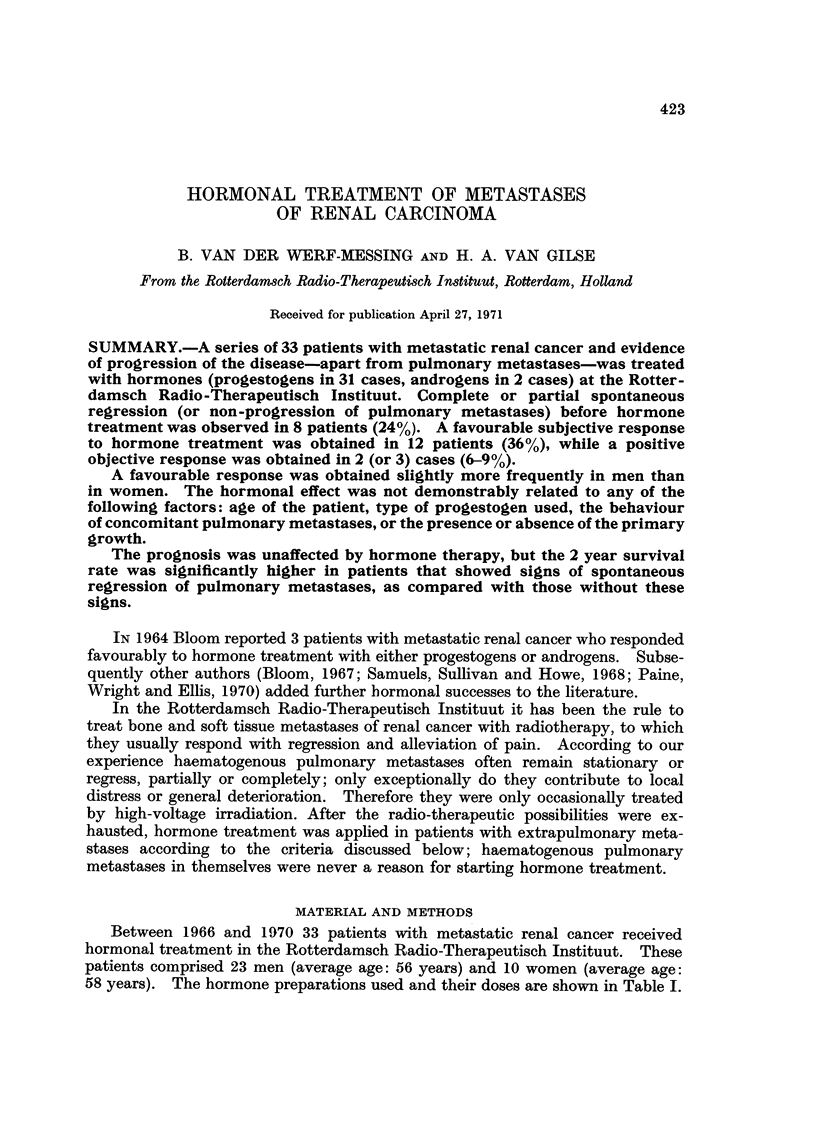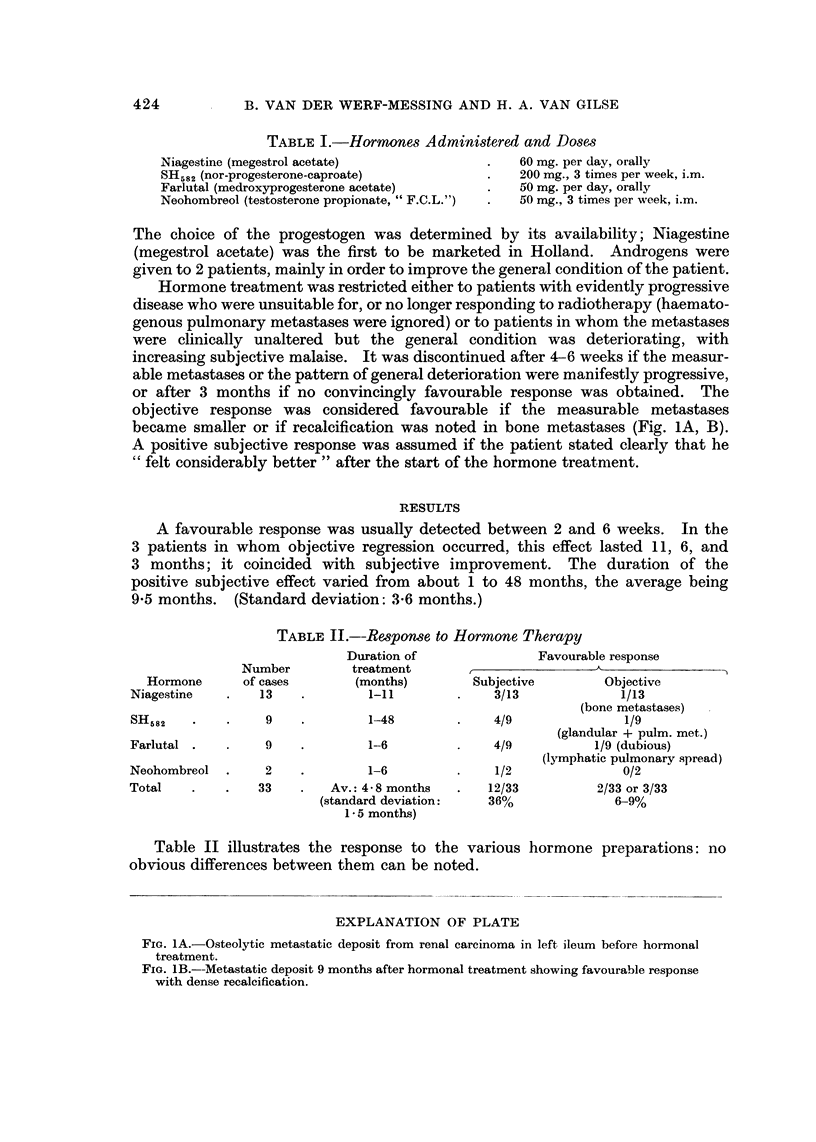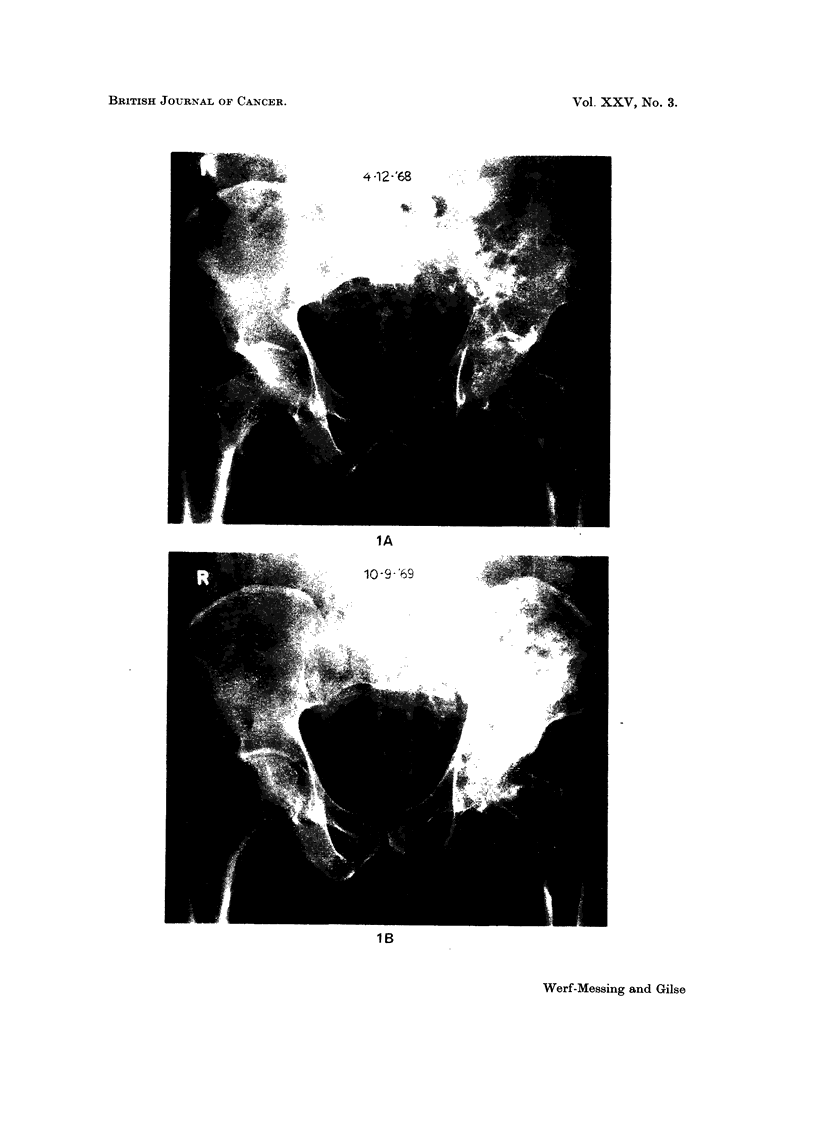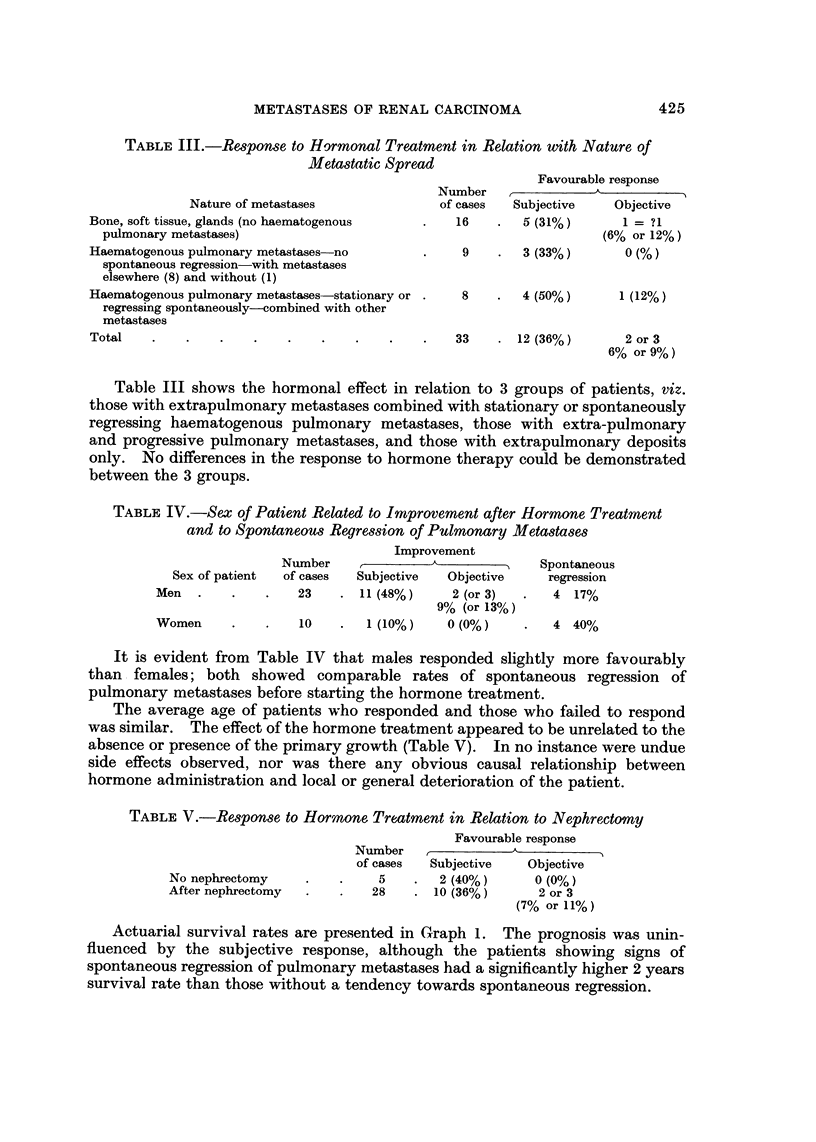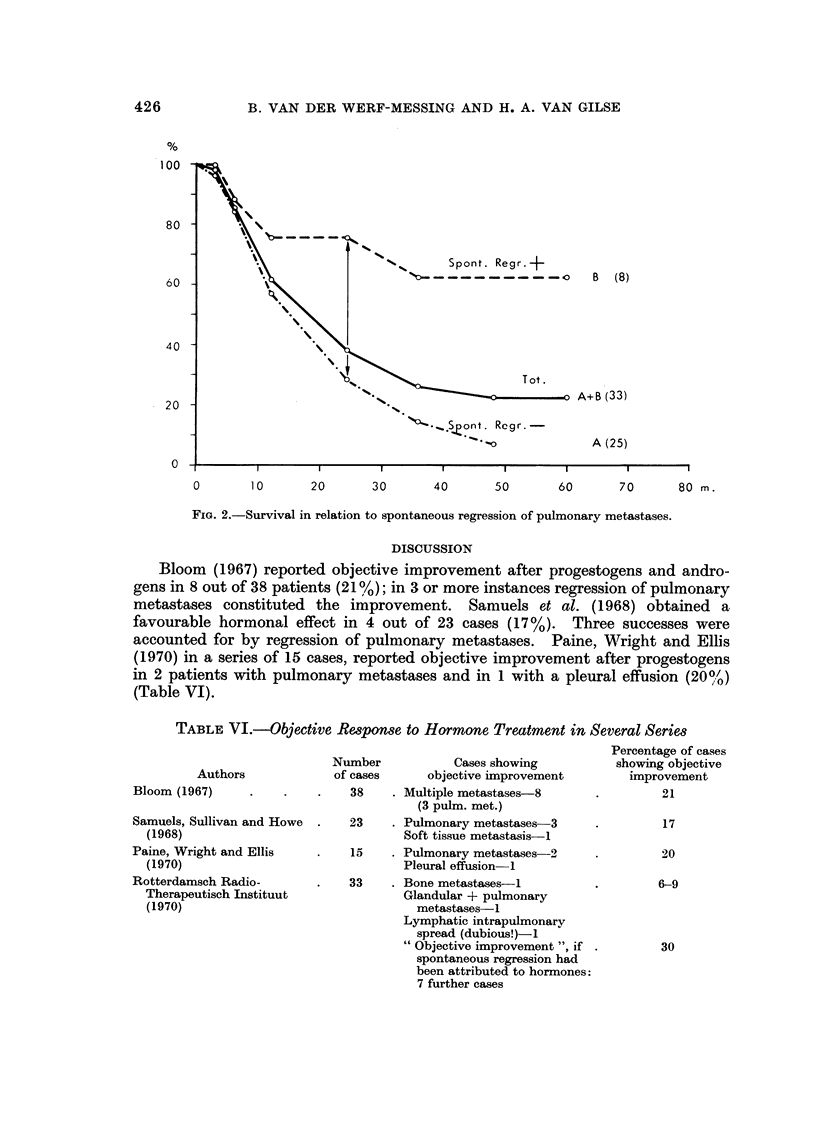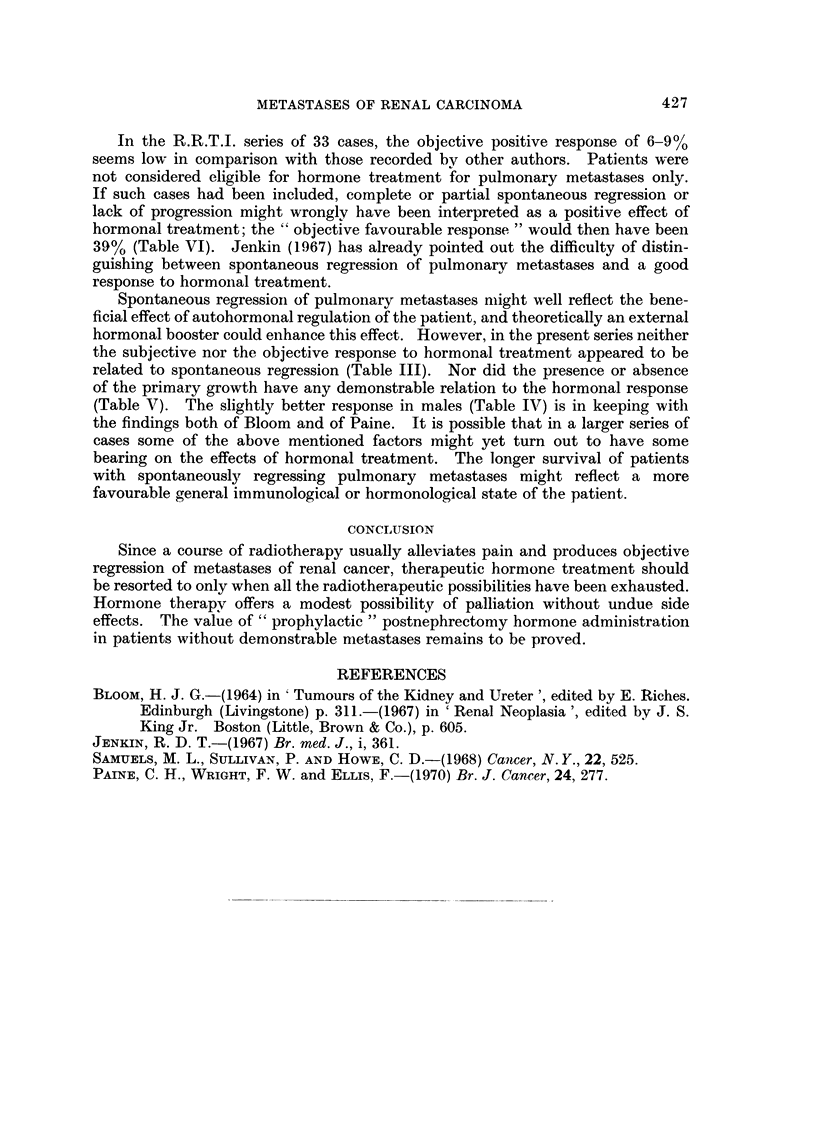# Hormonal Treatment of Metastases of Renal Carcinoma

**DOI:** 10.1038/bjc.1971.54

**Published:** 1971-09

**Authors:** B. van der Werf-Messing, H. A. van Gilse

## Abstract

**Images:**


### HORMONAL TREATMENT OF METASTASES OF RENAL CARCINOMA

B. VAN DER WERF-MESSING AND H. A. VAN GILSE

*From the Rotterdamsch Radio-Therapeutisch Instituut, Rotterdam, Holland*

Received for publication April 27, 1971

**SUMMARY.—A series of 33 patients with metastatic renal cancer and evidence of progression of the disease—apart from pulmonary metastases—was treated with hormones (progestogens in 31 cases, androgens in 2 cases) at the Rotterdamsch Radio-Therapeutisch Instituut. Complete or partial spontaneous regression (or non-progression of pulmonary metastases) before hormone treatment was observed in 8 patients (24%). A favourable subjective response to hormone treatment was obtained in 12 patients (36%), while a positive objective response was obtained in 2 (or 3) cases (6–9%).**

**A favourable response was obtained slightly more frequently in men than in women. The hormonal effect was not demonstrably related to any of the following factors: age of the patient, type of progestogen used, the behaviour of concomitant pulmonary metastases, or the presence or absence of the primary growth.**

**The prognosis was unaffected by hormone therapy, but the 2 year survival rate was significantly higher in patients that showed signs of spontaneous regression of pulmonary metastases, as compared with those without these signs.**

IN 1964 Bloom reported 3 patients with metastatic renal cancer who responded favourably to hormone treatment with either progestogens or androgens. Subsequently other authors (Bloom, 1967; Samuels, Sullivan and Howe, 1968; Paine, Wright and Ellis, 1970) added further hormonal successes to the literature.

In the Rotterdamsch Radio-Therapeutisch Instituut it has been the rule to treat bone and soft tissue metastases of renal cancer with radiotherapy, to which they usually respond with regression and alleviation of pain. According to our experience haematogenous pulmonary metastases often remain stationary or regress, partially or completely; only exceptionally do they contribute to local distress or general deterioration. Therefore they were only occasionally treated by high-voltage irradiation. After the radio-therapeutic possibilities were exhausted, hormone treatment was applied in patients with extrapulmonary metastases according to the criteria discussed below; haematogenous pulmonary metastases in themselves were never a reason for starting hormone treatment.

### MATERIAL AND METHODS

Between 1966 and 1970 33 patients with metastatic renal cancer received hormonal treatment in the Rotterdamsch Radio-Therapeutisch Instituut. These patients comprised 23 men (average age: 56 years) and 10 women (average age: 58 years). The hormone preparations used and their doses are shown in Table I.

TABLE I.—*Hormones Administered and Doses*

| | |
|---|---|
| Niagestine (megestrol acetate) | 60 mg. per day, orally |
| $SH_{582}$ (nor-progesterone-caproate) | 200 mg., 3 times per week, i.m. |
| Farlutal (medroxyprogesterone acetate) | 50 mg. per day, orally |
| Neohombreol (testosterone propionate, " F.C.L.") | 50 mg., 3 times per week, i.m. |

The choice of the progestogen was determined by its availability; Niagestine (megestrol acetate) was the first to be marketed in Holland. Androgens were given to 2 patients, mainly in order to improve the general condition of the patient.

Hormone treatment was restricted either to patients with evidently progressive disease who were unsuitable for, or no longer responding to radiotherapy (haematogenous pulmonary metastases were ignored) or to patients in whom the metastases were clinically unaltered but the general condition was deteriorating, with increasing subjective malaise. It was discontinued after 4–6 weeks if the measurable metastases or the pattern of general deterioration were manifestly progressive, or after 3 months if no convincingly favourable response was obtained. The objective response was considered favourable if the measurable metastases became smaller or if recalcification was noted in bone metastases (Fig. 1A, B). A positive subjective response was assumed if the patient stated clearly that he " felt considerably better " after the start of the hormone treatment.

### RESULTS

A favourable response was usually detected between 2 and 6 weeks. In the 3 patients in whom objective regression occurred, this effect lasted 11, 6, and 3 months; it coincided with subjective improvement. The duration of the positive subjective effect varied from about 1 to 48 months, the average being 9·5 months. (Standard deviation: 3·6 months.)

TABLE II.—*Response to Hormone Therapy*

| Hormone | Number of cases | Duration of treatment (months) | Favourable response: Subjective | Favourable response: Objective |
|---|---|---|---|---|
| Niagestine | 13 | 1–11 | 3/13 | 1/13 (bone metastases) |
| $SH_{582}$ | 9 | 1–48 | 4/9 | 1/9 (glandular + pulm. met.) |
| Farlutal | 9 | 1–6 | 4/9 | 1/9 (dubious) (lymphatic pulmonary spread) |
| Neohombreol | 2 | 1–6 | 1/2 | 0/2 |
| Total | 33 | Av.: 4·8 months (standard deviation: 1·5 months) | 12/33 36% | 2/33 or 3/33 6–9% |

Table II illustrates the response to the various hormone preparations: no obvious differences between them can be noted.

---

### EXPLANATION OF PLATE

FIG. 1A.—Osteolytic metastatic deposit from renal carcinoma in left ileum before hormonal treatment.

FIG. 1B.—Metastatic deposit 9 months after hormonal treatment showing favourable response with dense recalcification.

1A

1B

Werf-Messing and Gilse

TABLE III.—*Response to Hormonal Treatment in Relation with Nature of Metastatic Spread*

| Nature of metastases | Number of cases | Favourable response Subjective | Objective |
|---|---|---|---|
| Bone, soft tissue, glands (no haematogenous pulmonary metastases) | 16 | 5 (31%) | 1 = ?1 (6% or 12%) |
| Haematogenous pulmonary metastases—no spontaneous regression—with metastases elsewhere (8) and without (1) | 9 | 3 (33%) | 0 (%) |
| Haematogenous pulmonary metastases—stationary or regressing spontaneously—combined with other metastases | 8 | 4 (50%) | 1 (12%) |
| Total | 33 | 12 (36%) | 2 or 3 6% or 9%) |

Table III shows the hormonal effect in relation to 3 groups of patients, *viz.* those with extrapulmonary metastases combined with stationary or spontaneously regressing haematogenous pulmonary metastases, those with extra-pulmonary and progressive pulmonary metastases, and those with extrapulmonary deposits only. No differences in the response to hormone therapy could be demonstrated between the 3 groups.

TABLE IV.—*Sex of Patient Related to Improvement after Hormone Treatment and to Spontaneous Regression of Pulmonary Metastases*

| Sex of patient | Number of cases | Improvement Subjective | Objective | Spontaneous regression |
|---|---|---|---|---|
| Men | 23 | 11 (48%) | 2 (or 3) 9% (or 13%) | 4 17% |
| Women | 10 | 1 (10%) | 0 (0%) | 4 40% |

It is evident from Table IV that males responded slightly more favourably than females; both showed comparable rates of spontaneous regression of pulmonary metastases before starting the hormone treatment.

The average age of patients who responded and those who failed to respond was similar. The effect of the hormone treatment appeared to be unrelated to the absence or presence of the primary growth (Table V). In no instance were undue side effects observed, nor was there any obvious causal relationship between hormone administration and local or general deterioration of the patient.

TABLE V.—*Response to Hormone Treatment in Relation to Nephrectomy*

| | Number of cases | Favourable response Subjective | Objective |
|---|---|---|---|
| No nephrectomy | 5 | 2 (40%) | 0 (0%) |
| After nephrectomy | 28 | 10 (36%) | 2 or 3 (7% or 11%) |

Actuarial survival rates are presented in Graph 1. The prognosis was uninfluenced by the subjective response, although the patients showing signs of spontaneous regression of pulmonary metastases had a significantly higher 2 years survival rate than those without a tendency towards spontaneous regression.

FIG. 2.—Survival in relation to spontaneous regression of pulmonary metastases.

### DISCUSSION

Bloom (1967) reported objective improvement after progestogens and androgens in 8 out of 38 patients (21%); in 3 or more instances regression of pulmonary metastases constituted the improvement. Samuels *et al.* (1968) obtained a favourable hormonal effect in 4 out of 23 cases (17%). Three successes were accounted for by regression of pulmonary metastases. Paine, Wright and Ellis (1970) in a series of 15 cases, reported objective improvement after progestogens in 2 patients with pulmonary metastases and in 1 with a pleural effusion (20%) (Table VI).

TABLE VI.—*Objective Response to Hormone Treatment in Several Series*

| Authors | Number of cases | Cases showing objective improvement | Percentage of cases showing objective improvement |
|---|---|---|---|
| Bloom (1967) | 38 | Multiple metastases—8 (3 pulm. met.) | 21 |
| Samuels, Sullivan and Howe (1968) | 23 | Pulmonary metastases—3<br>Soft tissue metastasis—1 | 17 |
| Paine, Wright and Ellis (1970) | 15 | Pulmonary metastases—2<br>Pleural effusion—1 | 20 |
| Rotterdamsch Radio-Therapeutisch Instituut (1970) | 33 | Bone metastases—1<br>Glandular + pulmonary metastases—1<br>Lymphatic intrapulmonary spread (dubious!)—1 | 6–9 |
| | | " Objective improvement ", if spontaneous regression had been attributed to hormones: 7 further cases | 30 |

In the R.R.T.I. series of 33 cases, the objective positive response of 6–9% seems low in comparison with those recorded by other authors. Patients were not considered eligible for hormone treatment for pulmonary metastases only. If such cases had been included, complete or partial spontaneous regression or lack of progression might wrongly have been interpreted as a positive effect of hormonal treatment; the " objective favourable response " would then have been 39% (Table VI). Jenkin (1967) has already pointed out the difficulty of distinguishing between spontaneous regression of pulmonary metastases and a good response to hormonal treatment.

Spontaneous regression of pulmonary metastases might well reflect the beneficial effect of autohormonal regulation of the patient, and theoretically an external hormonal booster could enhance this effect. However, in the present series neither the subjective nor the objective response to hormonal treatment appeared to be related to spontaneous regression (Table III). Nor did the presence or absence of the primary growth have any demonstrable relation to the hormonal response (Table V). The slightly better response in males (Table IV) is in keeping with the findings both of Bloom and of Paine. It is possible that in a larger series of cases some of the above mentioned factors might yet turn out to have some bearing on the effects of hormonal treatment. The longer survival of patients with spontaneously regressing pulmonary metastases might reflect a more favourable general immunological or hormonological state of the patient.

### CONCLUSION

Since a course of radiotherapy usually alleviates pain and produces objective regression of metastases of renal cancer, therapeutic hormone treatment should be resorted to only when all the radiotherapeutic possibilities have been exhausted. Hormone therapy offers a modest possibility of palliation without undue side effects. The value of " prophylactic " postnephrectomy hormone administration in patients without demonstrable metastases remains to be proved.

### REFERENCES

Bloom, H. J. G.—(1964) in ' Tumours of the Kidney and Ureter ', edited by E. Riches. Edinburgh (Livingstone) p. 311.—(1967) in ' Renal Neoplasia ', edited by J. S. King Jr. Boston (Little, Brown & Co.), p. 605.

Jenkin, R. D. T.—(1967) *Br. med. J.*, i, 361.

Samuels, M. L., Sullivan, P. and Howe, C. D.—(1968) *Cancer, N.Y.*, **22**, 525.

Paine, C. H., Wright, F. W. and Ellis, F.—(1970) *Br. J. Cancer*, **24**, 277.